# Quantification of fairness bias by a Fairness-Equity Model

**DOI:** 10.1186/1471-2202-12-S1-P327

**Published:** 2011-07-18

**Authors:** David Nicoladie Tam

**Affiliations:** 1 Department of Biological Sciences, University of North Texas, Denton, TX 76203, USA

## 

We have developed a computational model that quantifies fairness objectively based on equity/disparity between the shares by two individuals. This Fairness-Equity Model [1] represents the relationship between fairness perception intensity and the disparity (or equity) between two individuals graphically. Fairness bias is represented by what is considered as equitable between the shares. Absolute equity represents equal shares, while relative equity represents unequal shares. Tolerance to inequity is represented by the unequal share, yet considered as fair. This represents fairness bias, in which inequitable share is tolerated as fair. This tolerance for inequity can be represented graphically by the fairness-equity graph by shifting the fairness-curve horizontally. Sensitivity to fairness or unfairness can also be represented by the increasing the slope of the fairness-curve. Fairness perception is also hypothesized by the proportionality relationship to the equity/disparity measures.

Confirmation of this proportional hypothesis is provided by using the Ultimatum Game (UG) with human subjects. The results show that fairness perception is proportional to the equity/disparity signal. The fairness biases are also quantified the shifting of the fairness-curve relative to the acceptance and rejection decision in the UG experiment. It further reveals the objectivity of fairness perception to equitable share compared to the subjectivity of fairness perception to inequitable share.
